# A virulent *Bacillus cereus* strain from deep-sea cold seep induces pyroptosis in a manner that involves NLRP3 inflammasome, JNK pathway, and lysosomal rupture

**DOI:** 10.1080/21505594.2021.1926649

**Published:** 2021-05-19

**Authors:** Yan Zhao, Shuai Jiang, Jian Zhang, Xiao-Lu Guan, Bo-Guang Sun, Li Sun

**Affiliations:** a College of Earth and Planetary Sciences, University of Chinese Academy of Sciences, Beijing, China; b Laboratory for Marine Biology and Biotechnology, Pilot National Laboratory for Marine Science and Technology (Qingdao), Qingdao, China; c CAS and Shandong Province Key Laboratory of Experimental Marine Biology, Institute of Oceanology, Center for Ocean Mega-Science, Chinese Academy of Sciences, Qingdao, China; d Deep Sea Research Center, Institute of Oceanology, Chinese Academy of Sciences, Qingdao, China

**Keywords:** *Bacillus cereus*, deep-sea cold seep, infection, NLRP3 inflammasome, pyroptosis

## Abstract

Recent studies indicate that the *Bacillus* species is distributed in deep-sea environments. However, no specific studies on deep-sea *Bacillus cereus* have been documented. In the present work, we isolated a *B. cereus* strain, H2, from the deep-sea cold seep in South China Sea. We characterized the pathogenic potential of H2 and investigated H2-induced death of different types of cells. We found that H2 was capable of tissue dissemination and causing acute mortality in mice and fish following intraperitoneal/intramuscular injection. *In vitro* studies revealed that H2 infection of macrophages induced pyroptosis and activation of the NLRP3 inflammasome pathway that contributed partly to cell death. H2 infection activated p38, JNK, and ERK, but only JNK proved to participate in H2-triggered cell death. Reactive oxygen species (ROS) and intracellular Ca^2+^ were essential to H2-induced activation of JNK and NLRP3 inflammasome. In contrast, lysosomal rupture and cathepsins were required for H2-induced NLRP3 inflammasome activation but not for JNK activation. This study revealed for the first time the virulence characteristics of deep-sea *B. cereus* and provided new insights into the mechanism of *B. cereus* infection.

## Introduction

*Bacillus cereus* is widespread in nature and frequently isolated from soil, plants, water, and food production system niches [1]. Recently, *B. cereus* was reported to exist in diverse marine environments including deep sea [2]. As a human pathogen, *B. cereus* was initially characterized as a causal agent of gastroenteritis, which was considered the second most important cause of food poisoning incidents in China, after *Salmonella* spp [1,3]. The number of cases of *B. cereus* food-borne infections is probably underreported due to several reasons [4]. *B. cereus* is also an opportunistic pathogen causing several severe non-gastrointestinal infections such as sepsis, endophthalmitis, pneumonia, and endocarditis [5]. Various *B. cereus* exotoxins contribute to the pathogenicity for gastrointestinal infections and non-gastrointestinal infections associated with human diseases [6]. Studies have shown that *B. cereus* caused mitochondrial destabilization, activated caspase-8 and caspase-3, and induced apoptosis and inflammasome in several cell lines [7–12]. However, a clearer understanding of many aspects of the pathogenesis requires further investigation.

Pyroptosis is a type of programmed cell death mediated by the gasdermin protein family. It is characterized by swelling of the cells and formation of large bubbles from the plasma membrane [13,14]. Pyroptosis is commonly induced by the activation of canonical inflammasomes and noncanonical inflammasomes in the context of infection, tissue injury, or metabolic imbalances [15]. In caspase-1 (Casp1)-dependent canonical inflammasomes, Nod-like receptors (NLR; e.g., NLRP1, NLRP2, NLRP3, NLRP6, NLRP7, and NLRC4) and non-NLR (e.g., AIM2) can be selectively activated by pathogen-associated molecular patterns (PAMPs), damage-associated molecular patterns (DAMPs), or other immune stimulations, which leads to cleavage of pro-Casp1 to form activated Casp1 [16]. Caspase-11 (mouse) and caspase-4/5 (humans) dependent noncanonical inflammasome is activated by cytosolic bacterial lipopolysaccharide (LPS) in several types of cells [17]. Both activated Casp1 and Casp4/5/11 can cleave gasdermin D (GSDMD) into a 31 kDa N-terminal fragment and a 22 kDa C-terminal fragment, the former moves to the membrane and mediates membrane perforation, resulting in extracellular content infiltration and cell swelling [18–20]. The pyroptotic cells release the pro-inflammatory cytokines interleukin (IL)-1β and IL-18, which recruit inflammatory cells that subsequently induces inflammation [21]. To date, NLRP3-mediated inflammasome is most studied, due to its crucial involvement in various infection- and non-infection-triggered inflammation [22]. NLRP3 responds to cellular perturbations including lysosomal rupture [23–25], potassium efflux [26,27], calcium influx [28], changes in cell volume [29], mitochondrial dysfunction [27,30], reactive oxygen species (ROS) [27,31] and JNK activation [25,32]. However, the mechanism of how these stimuli activate NLRP3 is unclear.

Deep-sea cold seeps usually develop in continental margin sediments, where hydrocarbon-rich (primarily methane) and hydrogen sulfide fluids rise to the seafloor and support oasis ecosystems composed of various microorganisms and faunal assemblages [33–35]. The cold seeps in South China Sea (SCS) were first discovered in 2004 [36]. Subsequently, the population structure and phylogenetic diversity of the microbes in the cold seeps of SCS have been investigated [34,37,38]. In this study, we reported the first identification of a *B. cereus* strain, H2, from the deep-sea cold seep in SCS. We investigated the virulence feature of H2 and examined the immune response induced by H2 in various cells. We found that H2 can be lethal to both higher and lower vertebrates, and that H2 induced pyroptotic cell death and NLRP3 inflammasome activation in a manner that involved MAPK-JNK pathway and lysosomal rupture. These results provided new insights into the pathogenic potential and mechanism of deep-sea *B. cereus*.

## Materials and methods

### Reagents, media, kits, and antibodies

Dulbecco’s modified eagle medium (DMEM) and Roswell Park Memorial Institute (RPMI) 1640 medium were purchased from Corning (USA), fetal bovine serum (FBS), Opti-MEM medium and acridine orange (AO) were purchased from Thermo Fisher Scientific (USA). Ultrapure lipopolysaccharide (LPS) from *Escherichia coli* 0111:B4 was purchased from Invivogen (USA). Adenosine 5′-triphosphate (ATP), dimethyl sulfoxide (DMSO), phorbol 12-myristate 13-acetate (PMA), nigericin (Nig), N-acetyl-L-cysteine (NAC), 2′,7′-dichlorofluorescin diacetate (H_2_DCFDA), and 2-mercaptoethano were purchased from Sigma-Aldrich (USA). The inhibitors, i.e., diphenyliodonium (DPI), bafilomycin A1 (Baf-A1), MCC950, VX-765, and BAPTA-AM were purchased from Selleck (USA). CA-074 methyl ester (CA-074Me) was purchased from Cayman (USA). CytoTox 96® Non-Radioactive Cytotoxicity Assay kit was purchased from Promega (USA). Mouse and human IL-1β ELISA kit was purchased from Neobioscience (China) and ABclonal (China), respectively. Propidium iodide (PI) was purchased from Beyotime Biotechnology (China). Antibodies against p-p38, p-ERK, p-JNK, human GSDMD, human/mouse IL-1β and human caspase-1 (Casp1) were purchased from Cell Signaling Technologies (USA). Antibodies against mouse GSDMD and Casp1 were purchased from Abcam (UK). HRP-conjugated goat anti-mouse or rabbit IgG and antibodies against β-actin were purchased from Proteintech (USA).

### Isolation of H2

Strain H2 was isolated from the setae of the galatheid crabs *Shinkaia crosnieri* collected at a deep-sea cold seep in South China Sea (119°17′08.053′′E, 22°06′55.548′′N; depth 1120.3 m). The crabs were collected with a remotely operated vehicle (ROV) and thoroughly washed with sterile seawater. For bacterial isolation, the setae were homogenized in PBS, and the homogenate was plated on marine 2216E agar medium [39]. The plates were incubated at 28°C for 2 days under aerobic conditions, and the colonies on the plates were examined and purified. One of the purified colonies was named strain H2.

### Average nucleotide identity (ANI) and DNA-DNA hybridization (DDH) analysis

The genomic sequencing of H2 was performed as previously reported [40]. The genome sequence data of H2 have been deposited in GenBank under the accession number CP043966 – CP043968. The ANI was calculated using the EzBiocloud web service (https://www.ezbiocloud.net). DDH was conducted between strain H2 and other representative strains of *B. cereus* group and analyzed using genome-to-genome distance calculator (GGDC2.1; https://ggdc.dsmz.de/ggdc.php). All the predicted pairwise DDH values were obtained under the recommended Formula 2 [41,42]. The genome sequences of the *B. cereus* group strains used in DDH were downloaded from NCBI, and their accession numbers are shown in Table S1.

### Animals and ethics statement

Clinically healthy turbot (average 18 g) were purchased from a local fish farm and maintained at 20°C in aerated seawater. Prior to the experiment, the fish were acclimatized in the laboratory for 2 weeks and verified to be healthy as described previously [43]. BALB/c mice (female, 6–8 weeks, and 14 ± 2 g) were purchased from Qingdao Daren Fortune Animal Technology Co., Ltd (China). For tissue collection, fish were euthanized with tricaine methanesulfonate (Sigma, USA), and mice were anesthetized with ketamine (80 mg/kg) (Ketavet, Germany) as reported previously [44]. The live animal studies were approved by the Ethics Committee of Institute of Oceanology, Chinese Academy of Sciences.

### Cell lines and culture conditions

The human monocytic cell line THP-1 and murine macrophage cell line J774A.1 were obtained from China Infrastructure of Cell Lines Resource (China). The murine macrophage cell line RAW264.7 and the human epithelial cell line HeLa cells and HEK293T cells were purchased from American Tissue Culture Collection (ATCC) (USA). HeLa *Gsdmd* knockout (HeLa*^Gsdmd^*^−KO^) cells were kindly provided by Dr. Feng Shao (National Institute of Biological Sciences, Beijing, China). Null (control), defNLRP3 (*Nlrp3* knockdown), and defCasp1(*Casp1* knockdown) THP-1 cells were obtained from InvivoGen (USA) and cultured according to the manufacturer’s instructions. THP-1 cells were cultured at 37°C in 5% CO_2_ humidified incubator and maintained in complete RPMI 1640 medium supplemented with 10% (v/v) FBS, 1 mM L-glutamine, 50 nM 2-mercaptoethanol. THP-1 cells were differentiated into macrophages by treatment with 50–100 nM of PMA overnight at 37°C. J774A.1 cells, RAW264.7 cells, HeLa cells and HEK293T cells were grown at 37°C in 5% CO_2_ humidified incubator and maintained in complete DMEM medium supplemented with 10% (v/v) FBS. The relevant reagents and media used in cell culture are described in the above section entitled “Reagents, media, kits, and antibodies”.

### *Generation of* Gsdmd *knockout THP-1 cells (THP-1*^Gsgmd*-KO*^)

The THP-1*^Gsgmd^*^−KO^ was created based on the method reported previously [45]. To prepare lentiviral construct that delivers Cas9, HEK293T cells in 10-cm dishes were transfected with 12.5 μg plentiCas9-Blast (Addgene #52,962, USA), 7.5 μg psPAX2 (Addgene #12,260, USA) and 5 μg pMD2.G (Addgene #12,259, USA) using Lipofectamine 3000 (Invitrogen, USA) for 6 h. The culture medium was then changed to high-serum DMEM (20% FBS with 25 mM HEPES). After 48 h, the culture medium was collected and centrifuged at 1500 *g* for 10 min, then the medium containing lentiviral particles was filter through low-binding 0.22-μm filters (Merck, Ireland). To obtain THP-1 cells expressing Cas9 components, THP-1 cells were prepared in approximately 20% density and infected with lentiviral particles containing 5 μg/mL polybrene (Beyotime, China) for 36 h. The cells with stable Cas9 expression were selected by using 12 μg/mL blasticidin (Beyotime, China). The gRNA, which targets the sequence of 5ʹ-CTTCCACTTCTACGATGCCA-3ʹ, was cloned into the lentiGuide-Puro vector (Addgene #52,963, USA). The lentiviral construct of gRNA was prepared in HEK293T cells in the same way as that of the Cas9 lentiviral construct described above. THP-1 cells stably expressing Cas9 were further infected with the virus of gRNA transduction supplemented with 5 μg/mL polybrene. The cells were selected with 2 μg/mL puromycin (Sangon Biotech, China). After 8 days, single cells were isolated by serial dilution and expanded to establish clonal cell lines. The cells were then screened for knockout clones by immunoblotting. One selected clone (Figure S1) was verified by PCR sequencing.

### In vivo *infection and bacterial recovery from animal tissues*

H2 was cultured in Luria–Bertani (LB) medium overnight under aerobic conditions at 37°C, and then sub-cultured in fresh LB medium (1:50) to an OD_600_ of 0.8. The bacterial cells were collected by centrifugation and resuspended in PBS to different concentrations. Median lethal dose (LD50) was determined as reported previously [44]. *In vivo* infection in fish and mice was conducted as reported previously [44]. Briefly, turbot were injected intramuscularly (i.m.) with 100 μl H2 suspension or the same volume of PBS (control). Mice were injected intraperitoneally (i.p.) with 100 μl H2 suspension or the same volume of PBS (control). Bacterial dissemination in tissues was determined by plate count as follows. The tissues were homogenized in PBS, and homogenates were diluted in PBS. The dilutions were plated on LB agar plates, and the plates were incubated at 37°C for overnight. The colonies that appeared on the plates were counted. The genetic nature of the colonies was verified by PCR.

### In vitro *infection*

All *in vitro* infections were performed in Opti-MEM medium. To examine H2-induced LDH and/or IL-1β release, 8 × 10^4^ J774A.1 cells/well, 8 × 10^4^ RAW264.7 cells, 5 × 10^4^ HeLa cells, or 6 × 10^4^ differentiated THP-1 cells/well were seeded into 96-well plates. For immunoblot analysis, 1 × 10^6^ J774A.1 cells/well or 8 × 10^5^ PMA-differentiated THP-1 cells/well were seeded into 12-well plates. When necessary, cells were primed prior to infection for 4 h with 1 μg/mL LPS. Except where indicated otherwise, all infections were performed with a MOI of 0.5 and for 2 h. To examine the effects of inhibitors, the cells were pre-treated with one of the following compounds for 1 h before infection: 50 μM MCC950, 50 μM VX-765, 50 μM SP600125, 50 μM PD98056, 50 μM SB203580, 20 μM DPI, 20 mM NAC, 50 μM BAPTA-AM, 1 μM Baf-A1, and/or 40 μM CA-074Me. To examine the effect of ATP and nigericin, the cells were treated as above, with the replacement of H2 infection by treatment of 5 mM ATP for 1 hour or 20 μM nigericin for 2 h or other indicated time.

### Measurement of ROS

LPS-primed J774A.1 cells (as described above) were incubated with H_2_DCFDA (10 μM) for 45 min at 37°C in the dark. After incubation, the cells were washed with PBS and pretreated with or without the indicated inhibitor for 1 h. The cells were then stimulated with 5 mM ATP for 15 min or infected with H2 (MOI, 0.5) as above for 1 h. The cells were washed two times with PBS and suspended in PBS (5 × 10^5^ cells/mL). The ROS in the cells was detected using a flow cytometer (BD FACSAria II, USA) with excitation at 488 nm and emission at 525 nm. The data were then analyzed, during which forward and side scatter gates were established to exclude cellular debris and aggregates.

### Immunoblotting

The cell culture supernatant and cell lysate used for immunoblot were prepared as follows. The cell culture supernatant was collected and precipitated with 10% trichloroacetic acid (v/v) for 1 h on ice. The sample was subjected to centrifugation at 20,000 g for 30 min at 4°C. The protein pellet was washed with ice-cold acetone, air-dried, resuspended in SDS–PAGE sample buffer, and heated to 100°C for 10 min. Cell lysates were prepared by lysis in RIPA buffer and SDS–PAGE sample buffer containing protease and phosphatase inhibitor cocktail (Beyotime, China). The lysates were heated at 100°C for 10 min. The samples were subjected to SDS-PAGE. The proteins were then transferred to nitrocellulose membranes (GE Healthcare, USA) by electroblotting. The membranes were blocked in 5% BSA or 5% skimmed milk and incubated with primary antibodies at 4°C for overnight or at room temperature for 1.5–2 h. The membranes were then incubated with horseradish peroxidase-conjugated secondary antibody for 1 h. Immunoreactive proteins were detected with the ECL method and imaged with the GelDoc XR System (Bio-Rad, USA).

### Cytotoxicity assay and IL-1β measurement

Cell death was determined by measuring LDH release using CytoTox 96® Non-Radioactive Cytotoxicity Assay kit. IL-1β release was determined using IL-1β ELISA kit according to the manufacturer’s instructions. The assays were performed at least three times.

### Effect of H2 culture supernatant on cell death

Strain H2 was cultured in LB medium to an OD_600_ of 0.8. The bacterial cells were removed by centrifugation, and the supernatant was collected. The supernatant was filtered through low-binding 0.22-μm filters (Merck, Ireland). Some of the filtered supernatant was fractionated using Amicon Ultra-15 centrifugal filter units (Merck, Ireland) with 30 kDa or 100 kDa cutoff. The supernatant at different concentrations (1%, 5%, and 10% (v/v)) was added to J774A.1 cells. After incubation for 2 h, the cells were analyzed for cell death and caspase 1 and GSDMD cleavage as described above.

### Microscopy

For microscopy, J774A.1 cells were incubated with strain H2 (MOI, 0.5) for the different times in 35-mm culture dishes containing Opti-MEM medium. To examine the integrity of cellular structure, PI (2 μg/mL) was added to the culture. After incubation for 5 min, the cells were observed with a confocal microscope (Zeiss LSM 710, Germany). To examine lysosomal membrane permeability, cells were stained with Acridine Orange (2 μg/mL) at 37°C for 15 min and then washed with PBS. Images were captured using a confocal microscope (Zeiss LSM 710, Germany).

### Statistical analysis

Statistical analyses were carried out with GraphPad Prism 7 (Graph-Pad Software). Student’s *t* test was used for analysis of two groups, and one-way analysis of variance (ANOVA) was used for analysis of three or more groups. Statistical significance was defined as *p < 0.05, **p < 0.01.

## Results

### Isolation and phylogenetic classification of strain H2

Strain H2 was isolated from the setae of the galatheid crab *Shinkaia crosnieri* collected at a cold seep of South China Sea. Analysis of 16S rRNA sequence indicated that strain H2 was closely related to the *Bacillus* species in the *B. cereus* group. A more discriminatory analysis based on average nucleotide identity (ANI) and predicted DNA-DNA hybridization (DDH) indicated that H2 was most closely related to *B. cereus* ATCC 14,579 ^T^ and *B. thuringiensis* ATCC 10,792 ^T^ (Table S1), with the values of ANI and DDH between H2 and *B. cereus* ATCC 14579 ^T^ being 98.88% and 90.50%, respectively, and the values of ANI and DDH between H2 and *B. thuringiensis* ATCC 10792 ^T^ being 96.52% and 70.50%, respectively. The hypothesized species demarcation threshold values of ANI and DDH are 95% and 70%, respectively, however, which could not provide a clear separation between *B. cereus* and *B. thuringiensis* [46,47]. Nevertheless, since the values of both ANI and DDH between H2 and *B. cereus* ATCC 14579 ^T^ are higher than that between H2 and *B. thuringiensis* ATCC 10792 ^T^, and, moreover, the plasmid-borne genes of *cry* and *cyt* (encoding insecticidal crystal proteins), which are considered typical features of *B. thuringiensis* [6], were not found in the genome of H2, we proposed H2 to be a member of *B. cereus* and named the strain *B. cereus* H2.

### H2 is lethal to mice and fish and causes rapid pyroptosis-like cell death

**Figure 1.**
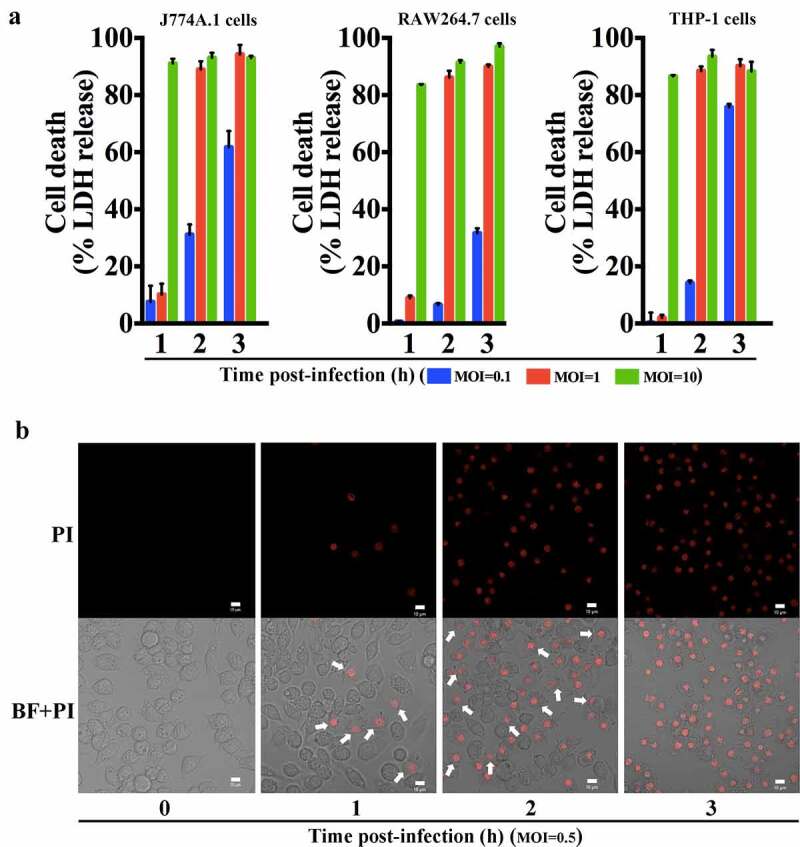
Strain H2 causes rapid death of macrophages. (a) J774A.1, RAW264.7, and PMA-differentiated THP-1 cells were infected with strain H2 at different MOI, and cell death was determined by measuring the release of lactate dehydrogenase (LDH) at different times post-infection. Data are the means of triplicate experiments and shown as means ± SD. (b) Representative images of J774A.1 cells uninfected (control) or infected with H2 (MOI = 0.5) for different hours. The cells were stained with propidium iodide (PI) and observed with a confocal microscope. Scale bar, 10 μm. Arrows indicate representative cells at different stages of death. The images are representative of three independent experiments

When H2 was inoculated into the teleost fish turbot via intramuscular (i.m.) injection, a LD_50_ of 4.4 × 10^5^ CFU was obtained. When inoculated into mice via intraperitoneal (i.p.) injection, H2 caused rapid mortality and exhibited a LD_50_ of 3.2 × 10^7^ CFU. At the dose of 8 × 10^7^ CFU, 100% mortality occurred within 24 h (Figure S2). Bacterial recovery analysis showed that following inoculation, H2 was detected in the liver, spleen, and kidney of the infected fish and mice (Figure S3). Cellular study showed that when J774A.1 cells (mice macrophages) were infected with H2, rapid and lytic cell death (reflected by LDH release) was observed in a manner that depended on the MOI (Figure 1(a)). Almost complete cell death was induced by H2 at 1 hpi when the MOI was 10 and at 2 hpi when the MOI was 1. Similar response to H2 infection was observed with other macrophage-derived cell lines, i.e., RAW264.7 cells and PMA-differentiated THP-1 cells (Figure 1(a)). Microscopy revealed that after H2 treatment, the cells exhibited pyroptosis-like death and became swollen/bubbled up and stainable by PI, and that the number of cells with these dying features increased with the time of infection (Figure 1(b)). To examine whether H2-mediated cell death was limited to phagocytic cells, H2 infection was performed with the epithelial cells HeLa. The results showed that rapid death of the cells occurred after H2 infection at an MOI of 1 and 10 (Figure S4A).

### H2 induces Casp1-mediated GSDMD cleavage and IL-1β maturation and secretion

**Figure 2.**
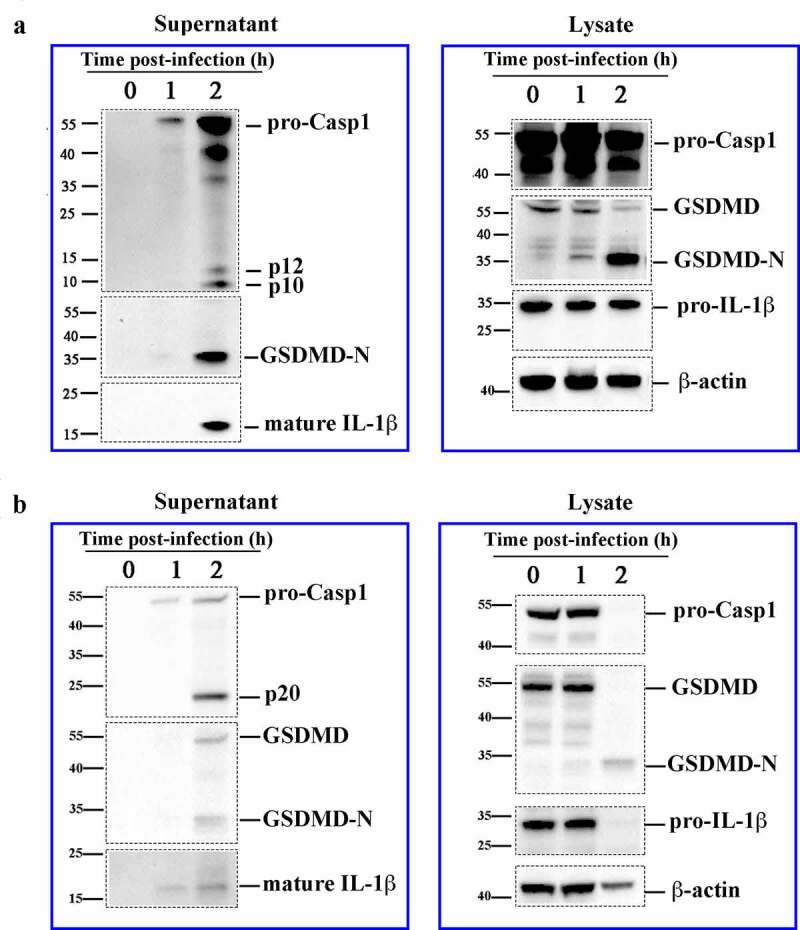
Strain H2 induces caspase-1 (Casp1) activation, GSDMD cleavage, and IL-1β maturation and secretion. J774A.1 cells (a) and PMA-differentiated THP-1 cells (b) were primed with LPS and infected with H2 (MOI = 1) for 1 h or 2 h. Cell lysate and culture supernatants were immunoblotted with antibodies against Casp1, GSDMD, IL-1β, or β-actin. β-actin was used as a loading control of cell lysate, the boundary of each blot was framed with dotted lines

Since, as shown above, H2 induced pyroptosis-like cell death, we investigated whether Casp1-mediated GSDMD cleavage was involved. The results showed that when LPS-primed J774A.1 cells were infected with H2, the p10 fragment of activated Casp1, the N terminal fragment of cleaved GSDMD (GSDMD-N) known as pyroptosis executor, and mature IL-1β were detected in the culture supernatant of the cells (Figure 2(a)). In the absence of LPS priming, H2 did not induce IL-1β synthesis, but still activated Casp1 and caused GSDMD cleavage (Figure S5). Similar to J774A.1 cells, when human PMA-differentiated THP-1 cells were primed with LPS and infected with H2, the p20 fragment of activated Casp1, GSDMD-N, and mature IL-1β were detected in the supernatant (Figure 2(b)). Compared to this observation in the wild type cells, H2-induced cell death and IL-1β release in the PMA-differentiated THP-1*^Gsdmd^*^−KO^ cells were markedly reduced, but still observable at a low level (Figure S6). Similar results were obtained with HeLa cells bearing *Gsdmd* knockout (Figure S4B). Collectively, these results indicate the existence of GSDMD–independent death mechanisms in H2-infected cells. Since the genome of H2 possesses the genes encoding the hemolysin BL (HBL) and non-hemolytic enterotoxin (NHE), which are well known extracellular cytotoxins [11,12], we examined whether the extracellular products of H2 had any effect on cell death. We found that the culture supernatant of H2 effectively triggered cell death, Casp1 activation, and GSDMD cleavage in a dose dependent manner (Figure S7A and B). Furthermore, size-fractionation of the supernatant revealed that the observed cell death was mediated by factors with molecular weights of >30 kDa and <100 kDa (Figure S7C).

### NLRP3 inflammasome activation is involved in H2-induced cell death

**Figure 3.**
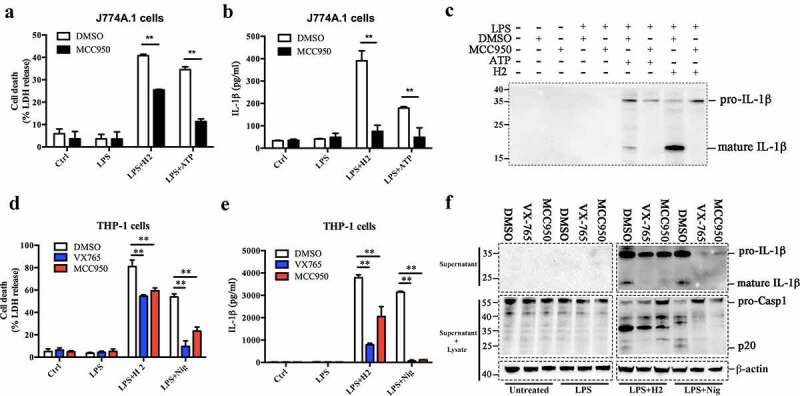
NLRP3 is involved in H2-induced cell death and IL-1β maturation and release. (a and b) J774A.1 cells were primed with or without (control) LPS and treated with MCC950 (NLRP3 inhibitor) or DMSO; the cells were then stimulated with ATP or infected with H2 (MOI = 0.5, 2 h). Supernatants of the cells were assayed for lactate dehydrogenase (LDH) (a) and IL-1β (b) release. (c) Supernatants from cells treated as above were immunoblotted with anti-IL-1β antibody. (d and e) PMA-differentiated THP-1 cells were primed with or without (control) LPS and treated with VX-765 (Casp1 inhibitor), MCC950, or DMSO; the cells were then stimulated with nigericin (Nig) or infected with H2 (MOI = 0.5, 2 h). Supernatants of the cells were assayed for LDH (d) and IL-1β (e) release. (f) PMA-differentiated THP-1 cells were treated as above in d and e. Culture supernatants were immunoblotted with anti-IL-1β antibody (upper panels); culture supernatants together with the corresponding cell lysates were immunoblotted with anti-Casp1 antibody and anti-β-actin antibody (middle and lower panels). β-actin was used as a loading control. Data are the representative of at least three independent experiments. For panels a, b, d, and e, data are the means of triplicate assay and shown as means ± SD. **p < 0.01, Student’s *t* test (a and b) or one-way ANOVA with Dunnett’s multiple-comparisons test (d and e). In panel c and f, the boundary of each blot was framed with dotted lines. Ctrl, control; DMSO, dimethyl sulfoxide, the dissolvent of the inhibitors

To gain insight into the mechanism of H2-induced cell death and Casp1 activation, we examined the potential involvement of NLRP3 in this process by using MCC950, a NLRP3-specific inhibitor that has no impact on AIM2, NLRP1, or NLRC4-mediated inflammation activation [48]. MCC950 significantly blocked ATP-triggered cell death and IL-1β secretion in LPS-primed J774A.1 cells (Figure 3(a-c)). Similarly, MCC950 significantly reduced H2-triggered cell death and IL-1β release from LPS-primed J774A.1 cells (Figure 3(a-c)). A time course analysis indicated that the effect of MCC950 was significant only at the relatively early infection time (2 h) and undetectable by 3 h of infection (Figure S8). In PMA-differentiated THP-1 cells, MCC950 and the Casp1-specific inhibitor VX-765 significantly suppressed H2-induced cell death and IL-1β release (Figure 3(d-f)). However, it should be noted that in both MCC950-treated J774A.1 cells and MCC950-treated THP-1 cells, substantial amounts of cell death still occurred. Immunoblot revealed that both MCC950 and VX-765 effectively blocked the cleavage of Casp1 in response to H2 infection and nigericin stimulation in PMA-differentiated THP-1 cells (Figure 3(f)). This observation was further confirmed by the study using the *Nlrp3* knockdown (defNLRP3) and *Casp1* knockdown (defCasp1) THP-1 cells, which showed that in these cells, H2-induced cell death and IL-1β release significantly decreased (Figure S9). Since potassium efflux is known to activate NLRP3 [15,22,26,27], we examined whether potassium had any effect on H2-induced cell death. The result showed that in PMA-differentiated THP-1 cells treated with H2, potassium supplementation significantly reduced LDH and IL-1β release in a dose-dependent manner (Figure S10). Taken together, these results indicated that the NLRP3 pathway was activated during H2 infection and significantly involved in cell death and, especially, IL-1β release, however, the activation of NLRP3 accounted only partly for the overall cell death induced by H2.

### JNK is crucial to H2-induced cell death

**Figure 4.**
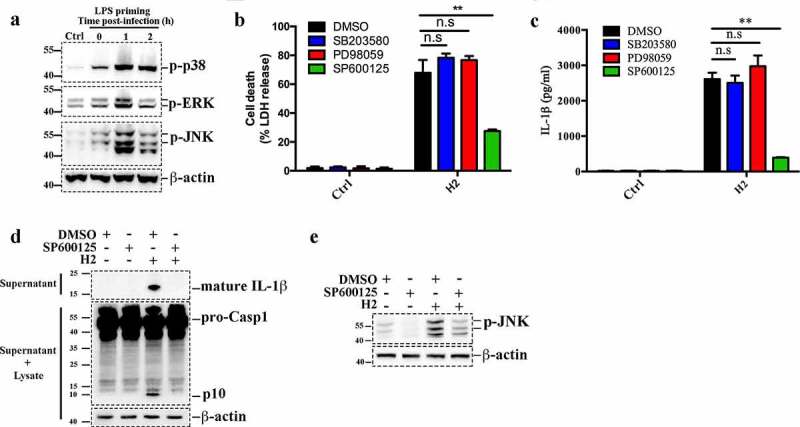
JNK plays a crucial role in H2-induced pyroptosis. (a) J774A.1 cells were primed with or without (control) LPS and then infected with H2 (MOI = 0.5) for different hours. Phosphorylation of p38, JNK, and ERK was determined by immunoblotting. β-actin was used as a loading control. (b, c) J774A.1 cells were primed with LPS and treated with p38 MAPK inhibitor SB203580, ERK inhibitor PD980659, JNK inhibitor SP600125, or DMSO for 1 h, and then infected with or without (control) H2 at MOI of 0.5 for 2 h. Supernatants of the cells were assayed for lactate dehydrogenase (LDH) (b) and IL-1β (c) release. (d) LPS-primed J774A.1 cells were treated with SP600125 or DMSO and then infected with H2 (MOI = 0.5, 2 h). Culture supernatants were immunoblotted with anti-IL-1β antibody (upper panels); culture supernatants together with the corresponding cell lysates were immunoblotted with anti-Casp1 antibody and anti-β-actin antibody (middle and lower panels). β-actin was used as a loading control. (e) Supernatants and cell lysates of (d) were subjected to immunoblot to detect JNK phosphorylation. For panels b and c, data are the means of triplicate assay and shown as means ± SD. **p < 0.01, one-way ANOVA with Dunnett’s multiple-comparisons test. In panels a, d, and e, the boundary of each blot was framed with dotted lines. Ctrl, control; DMSO, dimethyl sulfoxide, the dissolvent of the inhibitor. n.s, no significance

To examine the events upstream of NLRP3 inflammasome activation in H2-induced response, we investigated the involvement of MAPK signaling pathway in this process. The results showed that while LPS priming only moderately increased the phosphorylation levels of p38, ERK and JNK in J774A.1 cells, H2 infection markedly enhanced the phosphorylation of these proteins (Figure 4(a)). However, when p38, ERK and JNK were each inhibited by specific inhibitors, only inhibition of JNK significantly blocked H2-induced cell death and IL-1β release (Figure 4(b,c)). These results were confirmed by immunoblot, which showed that the JNK inhibitor SP600125 severely reduced JNK phosphorylation and abolished Casp1 activation and mature IL-1β release induced by H2 (Figure 4(d,e)).

### H2-induced JNK and NLRP3 activation is dependent on ROS production

**Figure 5.**
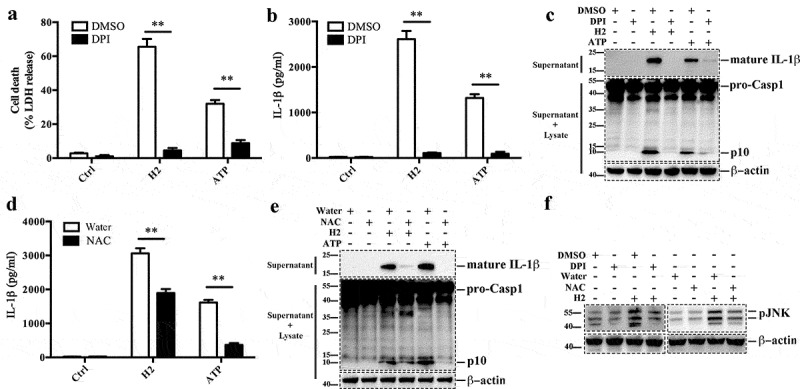
Strain H2-induced cell death and JNK activation are dependent on ROS production. (a and b) LPS-primed J774A.1 cells were pretreated with or without (control) diphenylene iodonium (DPI) or DMSO and then treated with or without strain H2 (MOI = 0.5, 2 h) or ATP. Supernatants were examined for release of lactate dehydrogenase (LDH) (a) and IL-1β (b). (c) LPS-primed J774A.1 cells were treated as above, supernatants were subjected to immunoblot to detect mature IL-1β (c, upper panel); supernatants plus cell lysates were subjected to immunoblot to detect caspase 1 (Casp1) activation (c, middle panel). (d) LPS-primed J774A.1 cells were pretreated with or without (control) N-acetyl-L-cysteine (NAC) or water (in which NAC was dissolved) and then treated with or without strain H2 (MOI = 0.5, 2 h) or ATP. Supernatants were examined for IL-1β release. (e) J774A.1 cells were treated as above in (d), Supernatants of the cells were subjected to immunoblot to detect mature IL-1β (e, upper panel); supernatants and cell lysates were subjected to immunoblot to detect Casp1 activation (e, middle panel). (f) Supernatants and cell lysates of (c, e) were mixed and subjected to immunoblot to detect JNK phosphorylation. In immunoblot assays, β-actin was used as a loading control. For panels a, b and d, data are the means of triplicate assay and shown as means ± SD. **p < 0.01, Student’s *t* test. Ctrl, control; DMSO, dimethyl sulfoxide (in which the inhibitor was dissolved)

Reactive oxygen species (ROS) is known to be an upstream activator of JNK pathway involved in NLRP3 inflammasome [49]. In this study, we found that the level of ROS in LPS-primed J774A.1 infected with strain H2 or stimulated with ATP was significantly increased (Figure S11). To investigate the potential involvement of ROS in H2-triggered cell death, we employed diphenyliodonium (DPI), an inhibitor of NADPH oxidase (NOX)-dependent ROS production as well as mitochondria-derived ROS production [50,51]. The results showed that DPI significantly reduced ATP-induced ROS production, cell death, and IL-1β secretion in J774A.1 (Figure 5(a-c), Figure S11A). Similarly, DPI significantly inhibited H2-triggered ROS production and completely blocked the death and IL-1β release of J774A.1 cells induced by H2 (Figure 5(a-c), Figure S11A). In addition, DPI blocked both ATP- and H2-induced Casp1 activation (Figure 5(c)). These observations were confirmed by the experiments using NAC, an antioxidant and a scavenger of ROS, which showed that, similar to DPI, NAC significantly reduced H2-mediated enhancement of ROS production, IL-1β release and Casp1 activation (Figure 5(d,e), Figure S11B). Furthermore, the presence of DPI or NAC inhibited JNK phosphorylation induced by H2 (Figure 5(f)). These results indicate that ROS production is essential to H2-triggered JNK and NLRP3 activation.

### Intracellular Ca^2+^ is required for H2-induced JNK activation and NLRP3 inflammasome

**Figure 6.**
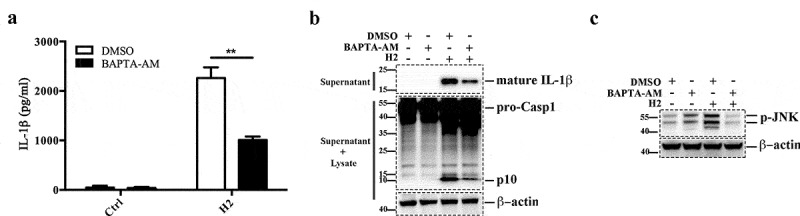
Strain H2 induces JNK activation is dependent on intracellular Ca^2+^ accumulation. (a) LPS-primed J774A.1 cells were pretreated with or without (control) DMSO or intracellular Ca^2+^ chelator BAPTA-AM and then infected with or without strain H2 (MOI = 0.5, 2 h), and IL-1β release in the supernatant of the cell culture was measured. Data are the means of triplicate assay and shown as means ± SD, **p < 0.01, Student’s *t* test. (b) LPS-primed J774A.1 cells were treated as above, supernatants were subjected to immunoblot to detect mature IL-1β (upper panel); supernatants plus cell lysates were subjected to immunoblot to detect caspase 1 (Casp1) activation (middle panel). (c) Supernatants and cell lysates of the cells of (b) were mixed and subjected to immunoblot to detect JNK phosphorylation. Ctrl, control; DMSO, dimethyl sulfoxide, the dissolvent of the inhibitor

Recent studies have shown that intracellular Ca^2+^ plays a critical role in the activation of JNK and subsequent induction of NLRP3 inflammasome activation [25,52]. We examined whether the intracellular Ca^2+^ chelator BAPTA-AM had any effect on H2-induced inflammasome activation. The results showed that BAPTA-AM significantly blocked H2-induced IL-1β secretion in J774A.1 cells (Figure 6(a,b)). Similarly, Casp1 activation and JNK phosphorylation induced by H2 were also markedly inhibited by BAPTA-AM (Figure 6(b,c)).

### H2-induced NLRP3 inflammasome, but not JNK, activation requires lysosomal rupture

**Figure 7.**
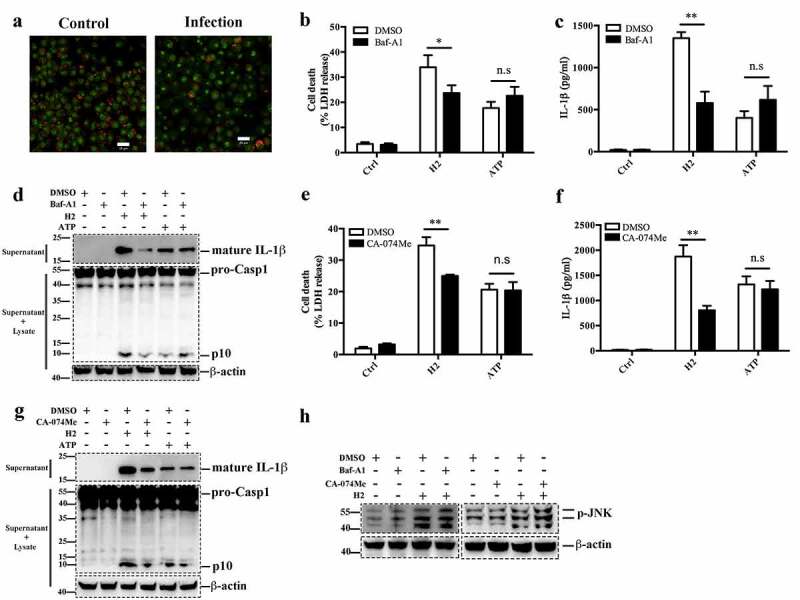
Lysosomal rupture is required for H2-induced NLRP3 but not for JNK activation. (a) LPS-primed J774A.1 cells were infected with or without (control) H2 (MOI = 0.5, 2 h) and stained with acridine orange (AO), the cells were then observed with a confocal microscope. AO fluoresces red in lysosome and green in the cytosol. Scale bar, 20 μm. (b, c) LPS-primed J774A.1 cells were pretreated with or without DMSO or Baf-A1 and then infected with or without (control) H2 or stimulated with ATP. Cell death was analyzed by lactate dehydrogenase (LDH) release (b), and IL-1β release was analyzed by ELISA (c). (d) J774A.1 cells were treated as (b, c), mature IL-1β (d, upper panel) and activation of caspase-1 (Casp1) (d, middle panel) were analyzed by Western blot. β-actin was used as loading control. (e-g) J774A.1 cells were treated as above (b-d), but in the presence of the cathepsin inhibitor CA-074Me, cell death (e), IL-1β release (f), and mature IL-1β and Casp1 (g) were determined as above. (h) J774A.1 cells were treated as (d, g), JNK phosphorylation were analyzed by Western blot. For panels B, C, E and F, data are the means of triplicate assay and shown as means ± SD. *p < 0.05; **p < 0.01, Student’s *t* test. Ctrl, control; DMSO, dimethyl sulfoxide, the dissolvent of the inhibitor. n.s, no significance

To investigate the potential involvement of lysosome destabilization in the activation of JNK by H2, we examined whether H2 infection could induce lysosome rupture. Confocal microscopy showed that in J774A.1 cells infected with H2 and stained with acridine orange (AO), cytosolic red fluorescence was markedly reduced compared to non-infected cells (Figure 7(a)), suggesting that H2 caused lysosomal damage. When the vacuolar H^+^-ATPase inhibitor bafilomycin-A1 (Baf-A1) was used to block the acidification and proteolytic function of lysosome, H2-triggered IL-1β release was significantly reduced in LPS-primed J774A.1 cells, but Baf-A1 had a moderate though significant effect on H2-triggered cell death (Figure 7(b-d)). Baf-A1 had no significant effect on ATP-induced cell death or IL-1β release. Consistently, Baf-A1 blocked Casp1 activation induced by H2 (Figure 7(d)). Furthermore, in the presence of the cathepsin family protein inhibitor CA-074Me, H2-triggered IL-1β release and Casp1 activation was significantly inhibited (Figure 7(e-g)). CA-074Me moderately though significantly decreased H2-triggered cell death (Figure 7(e)). However, neither Baf-A1 nor CA-074Me had apparent effect on H2-induced JNK phosphorylation (Figure 7(h)). These results suggest that lysosomal rupture and the ensuing release of functional lysosomal components, such as cathepsins, are essential to H2-induced inflammasome but not to H2-induced JNK activation.

## Discussion

Although *Bacillus* species have been reported to exist in various deep-sea environments [38,44,53,54], no studies on *B. cereus* from deep sea have been documented. Some *B. cereus* from land environments are known to be opportunistic pathogens that cause gastrointestinal diseases, such as diarrheal and emetic syndromes, and non-gastrointestinal infections, including sepsis, endophthalmitis, and pneumonia [1,4–6]. Several pioneering studies on deep-sea bacteria have indicated that some of these bacteria, such as *Vibrio, Sulfurovum, Caminibacter*, and *Nitratiruptor*, carry abundant virulence genes commonly found in non-marine bacterial pathogens, which raise the possibility that microbes isolated from deep sea might be pathogenic toward humans or animals on land [55–58]. In line with these predictions, we have in recent studies observed that *B. subtilis* and *B. wiedmannii* isolates from deep-sea hydrothermal vents were cytotoxic and lethal to lower and higher vertebrate animals [44,59]. In the present study, we found that another deep-sea isolate, *B. cereus* H2 from cold seep, was capable of tissue dissemination and inducing acute mortality in mice and fish, suggesting an active infectivity of this strain.

Pyroptosis is a type of programmed cell death that is mediated by gasdermins and results in rapid lytic cell death and the release of pro-inflammatory cytokines [14]. Pyroptosis can be triggered by a variety of stimuli, including invading pathogens [60,61]. In our study, H2 was found to induce rapid death of different types of mouse and human cells, including macrophages and epithelial cells, and H2-infected cells exhibited characteristics typical of that of pyroptosis, including membrane bubbling, GSDMD cleavage via Casp1, and release of IL-1β. Previous studies showed that although pyroptosis is often an advantageous process that clears invading pathogens to protect the host, excessive activation of the pytoptotic pathway can lead to lethal septic shock [62]. It is likely that the rapid death of H2-infected mice observed in our study was due at least in part to the rapid induction of pyroptosis-induced cell death caused by H2, which led to overwhelming inflammation that was fatal to the animal.

NLRP3 is a cytosolic sensor that responds to diverse stimuli and triggers pyroptosis [15]. In our study, we observed that inhibition or knockdown of NLRP3 markedly blocked H2-induced Casp1 activation and mature IL-1β secretion and also had a significant effect on cell death, indicating that H2 infection activated the NLRP3 inflammasome pathway. This is supported by the observation that K^+^, the outflow of which from the cytoplasm is known to activate NLRP3 [15,22,26,27], added to the cell culture system could prevent H2-induced cell death and IL-1β releases. However, it is noteworthy that inhibition of NLRP3 or Casp1 could only partially block LDH release, suggesting the presence of NLRP3- and Casp1-indepednent mechanisms of cell death in H2-infected cells. This hypothesis is in agreement with the observation that in both THP-1 and HeLa cells with *gsdmd* knockout, a certain amount of cell death could still occur following H2 infection. Recently, it was reported that the secreted hemolysin BL (HBL) and the non-hemolytic enterotoxin (NHE) of *B. cereus*, both are multi-component toxins of 30–50 kDa, activated inflammasome in macrophages via the K^+^ efflux-NLRP3 pathway [11,12]. Since the genome of H2 possesses the genes of HBL and NHE, H2 is endowed with the capacity to produce these toxins. Consistently, we found that the H2 culture supernatant fractions of 30–100 kDa were sufficient to kill host cells and activate Casp1 and GSDMD, suggesting that H2-induced cell death may be in part due to the action of the extracellular toxins. Previous studies have shown that the HBL and NHE toxins of *B. cereus* trigger NLRP3-dependent cell death at low concentration, but cause NLRP3-independent cell death at relatively high concentration (0.5 μM) [11,12]. In our study, we found that the significance of NLRP3 to H2-induced cell death was observed in the early, but not the late, infection hour. Since the extracellular toxins accumulate with time, they may cause massive NLRP3-independent cell death in the late stage of infection.

MAPK-JNK plays a vital role in various programmed cell deaths, including apoptosis, necroptosis, ferroptosis, and pyroptosis [63]. In our study, we observed that H2 infection induced cell death in macrophages through a pathway involving the stress-responsive MAPK-JNK. Further, we found that in addition to JNK, p38 and ERK were also activated by H2, but they did not participate in pyroptosis, indicating that p38 and ERK likely play a role different from that of JNK during H2 infection.

JNK signaling is activated by a wide range of factors, including ROS production, which is a required upstream event for NLRP3 activation [64–66]. Previous report showed that *B. cereus* infection could induce ROS production in Vero cells [10]. In our study, ROS production in H2-infected macrophages was significantly enhanced, and the presence of ROS inhibitors significantly reduced NLRP3 inflammasome activation and JNK phosphorylation. These results indicated that ROS was required for the pyroptosis triggered by H2. ROS is known to be generated from several sources, including mitochondria and NADPH oxidase [67]. A previous study showed that ROS generated by both mitochondria and NADPH oxidase were involved in JNK activation in *Vibrio cholerae* OmpU-treated macrophages [68]. In our study, we also observed an involvement of ROS in H2-induced JNK activation and pyroptosis. More importantly, we found that the inhibitory effect of DPI, which blocks ROS production by both NADPH oxidase and mitochondria, was much stronger than that of NAC, a ROS scavenger (Figure 5(b,d)). These observations suggested that, as reported previously [51], DPI probably affects mitochondrial functioning as well as ROS production. Since dysfunctioning mitochondria is known to induce NLRP3 inflammasome activation and JNK activation [14,63], the strong dramatic inhibitory effect of DPI observed in our study suggested an important role of mitochondria in H2-triggered pyroptosis.

Calcium signaling is a complex event that controls many cellular processes, including activation of NLRP3 inflammasome [69–72]. However, the precise mechanism of Ca^2+^ associated with the NLRP3 inflammasome pathway is largely unclear. Several works demonstrated that Ca^2+^ signaling modulates NLRP3 inflammasome activation by inducing MAPK pathways including JNK and ERK1/2 signaling [25,52,73]. It has been proposed that intracellular Ca^2+^ signaling triggers mitochondrial destabilization and subsequently generates mitochondrion-associated ligands that activate the NLRP3 inflammasome [71]. In our study, we found that the presence of the Ca^2+^ chelator BAPTA-AM significantly blocked H2-triggerd NLRP3 inflammasome activation and JNK phosphorylation in J774A.1 cells, indicating a link between intracellular Ca^2+^ signaling and H2-induced pyroptosis.

Lysosome participates in many cell death signaling in the context of apoptosis, autophagy-dependent cell death, ferroptosis, and pyroptosis [16,74,75]. It has been shown that lysosomal rupture liberates cathepsins, which subsequently activate the NLRP3 inflammasome [23,24,76]. One study demonstrated that the CaMKII/TAK1/JNK pathway was activated through lysosomal rupture and caused activation of the NLRP3 inflammasome [25]; another study showed that lysosomal rupture activated the NLRP3 inflammasome through the LAMP1/CaMKII/TAK1 signaling pathway [77]. However, the mechanism between lysosomal rupture and NLRP3 activation after *B. cereus* infection remained to be elucidated. In our work, we found that H2 infection caused lysosomal rupture in J774A.1 cells. Surprisingly, both Baf-A1 and CA-074Me markedly inhibited H2-induced inflammasome and IL-1β secretion but had no effect on JNK phosphorylation, suggesting that lysosomal rupture induced NLRP3 inflammasome activation in a manner that was independent of JNK activation.

In conclusion, we in this study demonstrated for the first time that virulent *B. cereus* exists in deep-sea cold seeps, and that the deep sea *B. cereus*, H2, induces host cell death and NLRP3 inflammasome activation in a manner that involves the JNK pathway and lysosomal rupture. Furthermore, our results suggest that H2 induces caspase 1- and GSDMD-independent cell death, and that NLRP3 is likely but one of the multiple players that take part in causing the death of host cells. These results provide new insights into the mechanism of *B. cereus* infection and *B. cereus*-induced host immune response.

## Supplementary Material

Supplemental Material

## Data Availability

The sequence data of this manuscript are available from GenBank under the accession number CP043966 - CP043968.
