# Supplementary figures and images for: A virulent Bacillus cereus strain from deep-sea cold seep induces pyroptosis in a manner that involves NLRP3 inflammasome, JNK pathway, and lysosomal rupture
Source: Virulence. 2021 May 19;12(1):1362–76. doi: 10.1080/21505594.2021.1926649 (PMC8143241; doi:10.1080/21505594.2021.1926649)

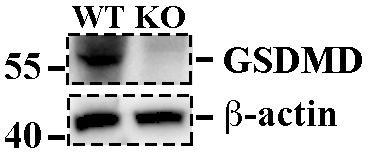

Supplement: Supplemental Material [file KVIR_A_1926649_SM3662.zip › supplementary(1926649)/Figure S1.tif]

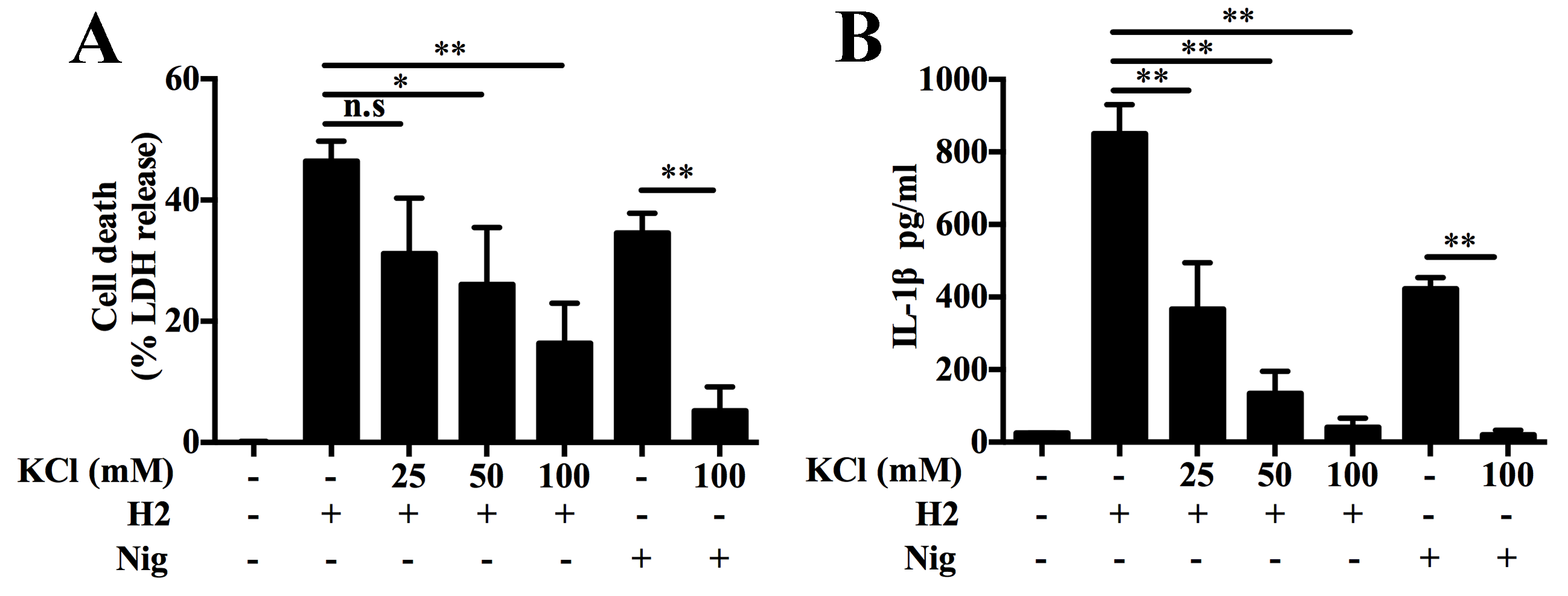

Supplement: Supplemental Material [file KVIR_A_1926649_SM3662.zip › supplementary(1926649)/Figure S10.tif]

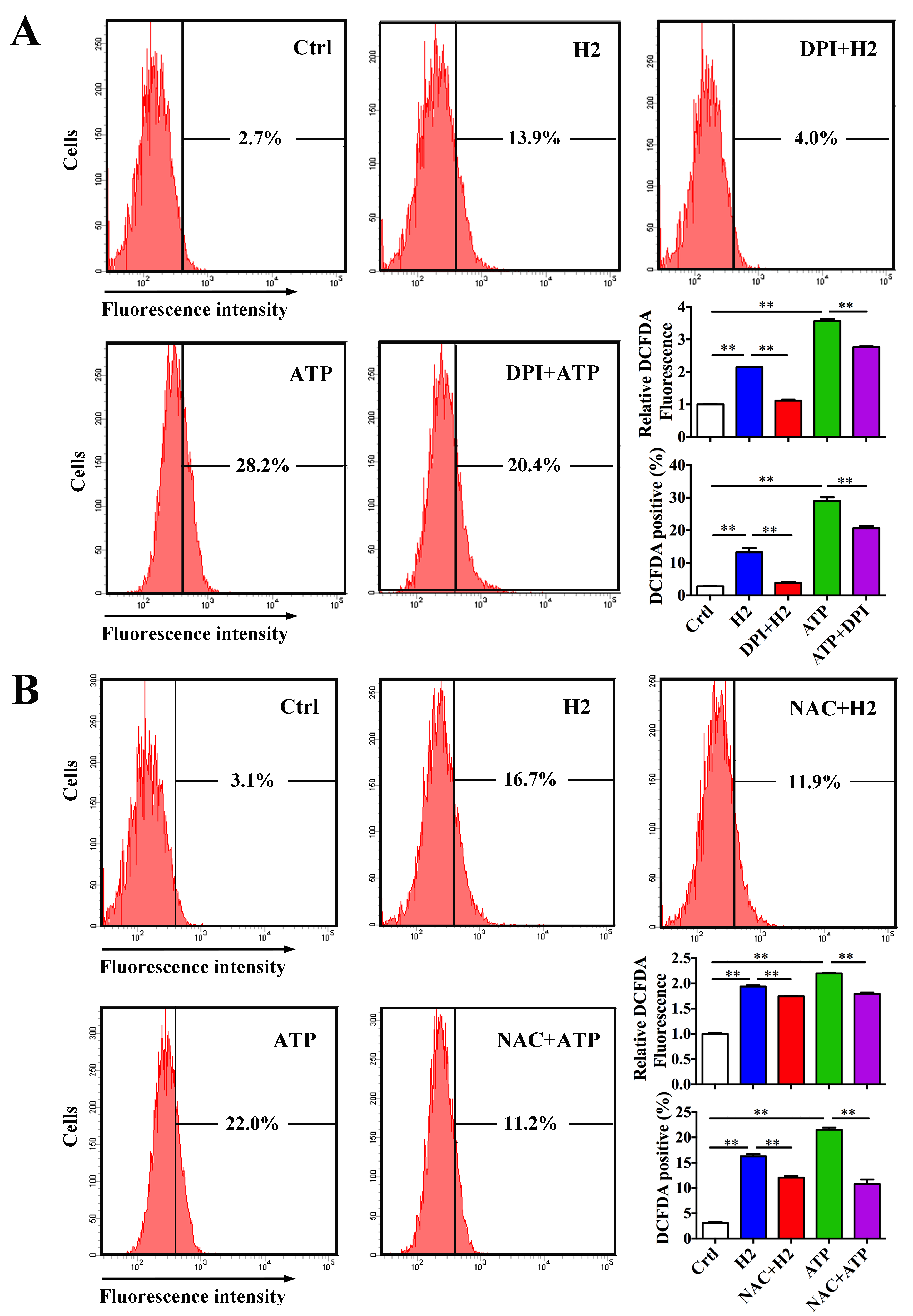

Supplement: Supplemental Material [file KVIR_A_1926649_SM3662.zip › supplementary(1926649)/Figure S11.tif]

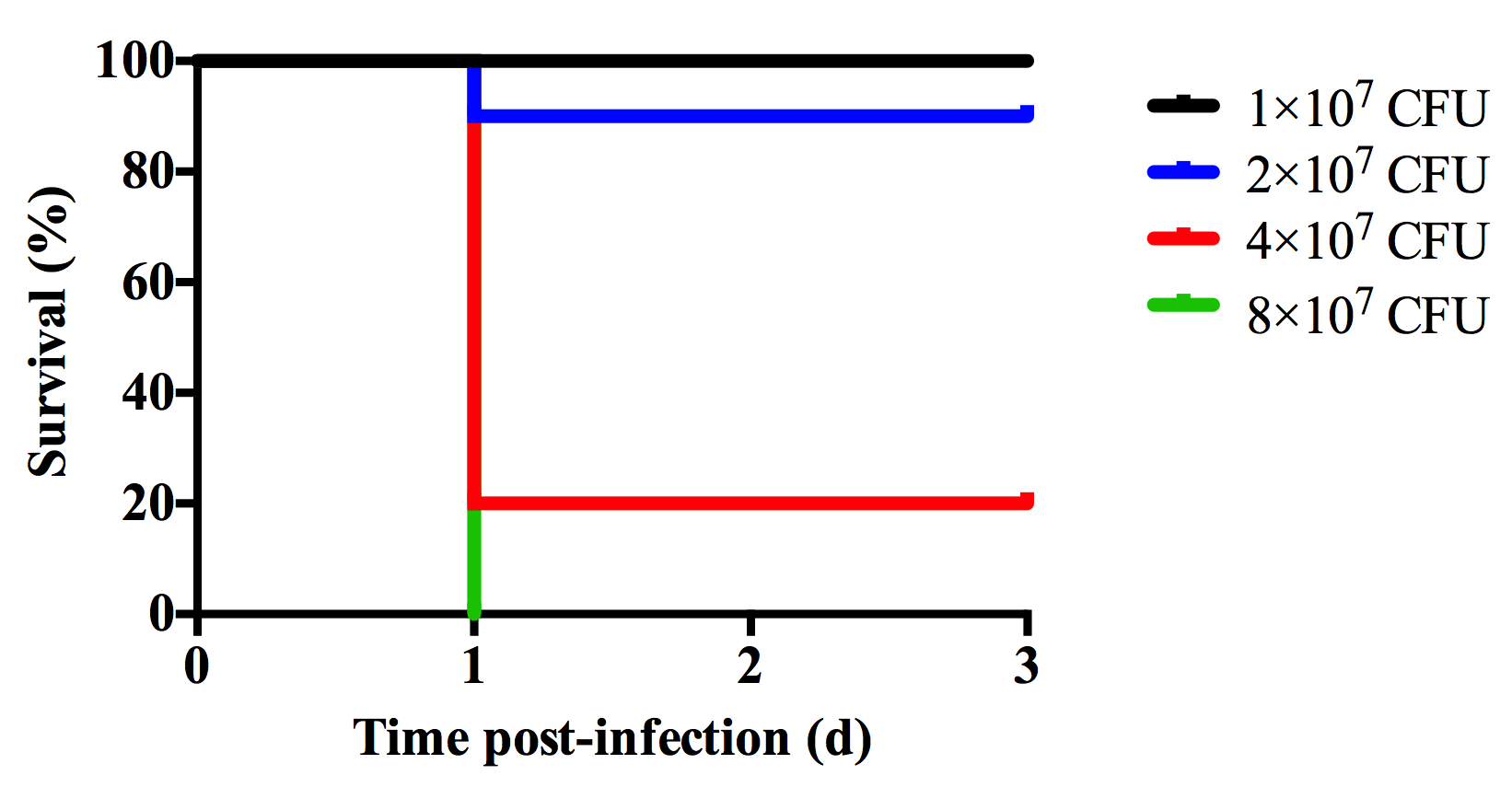

Supplement: Supplemental Material [file KVIR_A_1926649_SM3662.zip › supplementary(1926649)/Figure S2.tif]

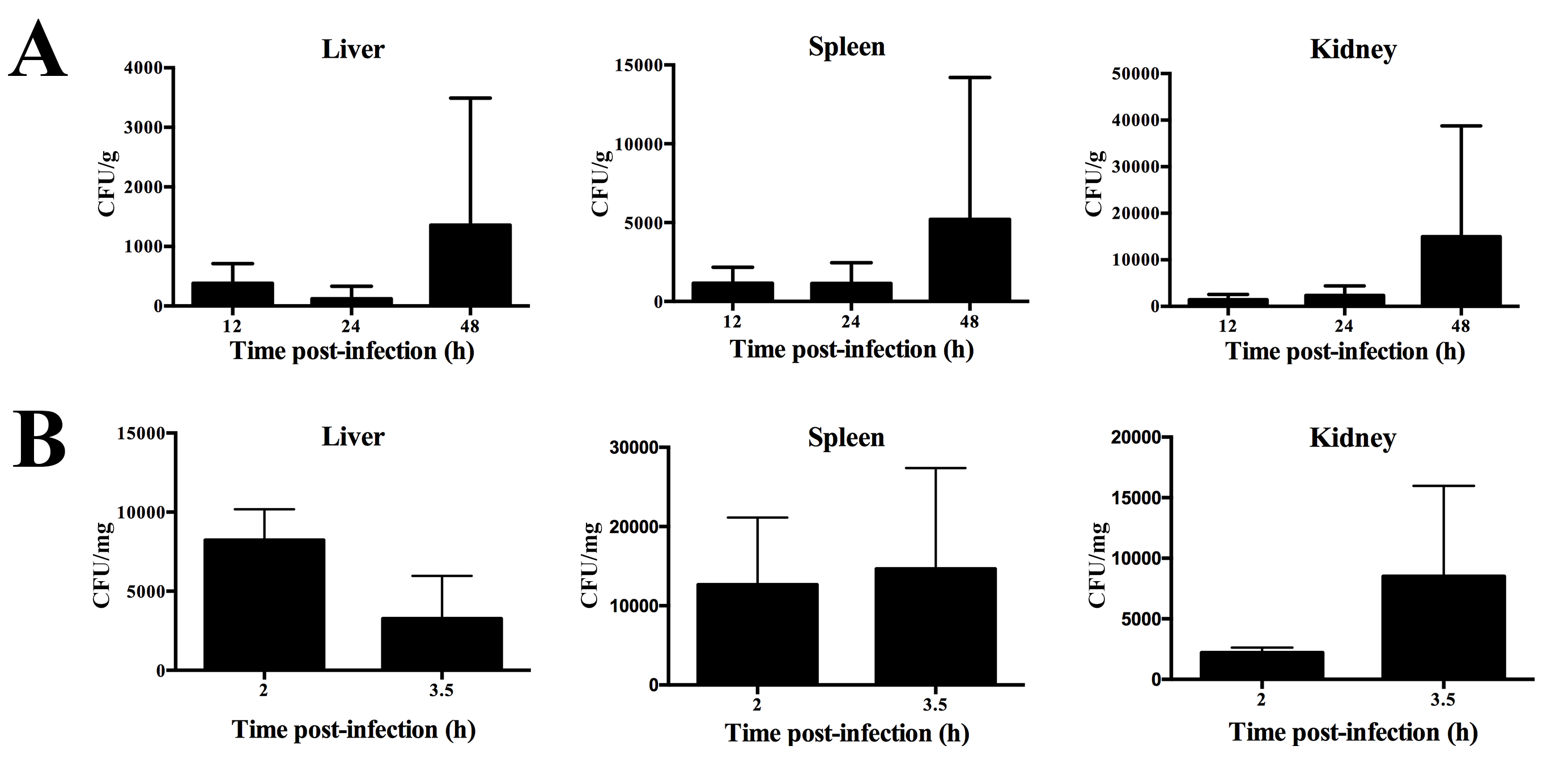

Supplement: Supplemental Material [file KVIR_A_1926649_SM3662.zip › supplementary(1926649)/Figure S3.tif]

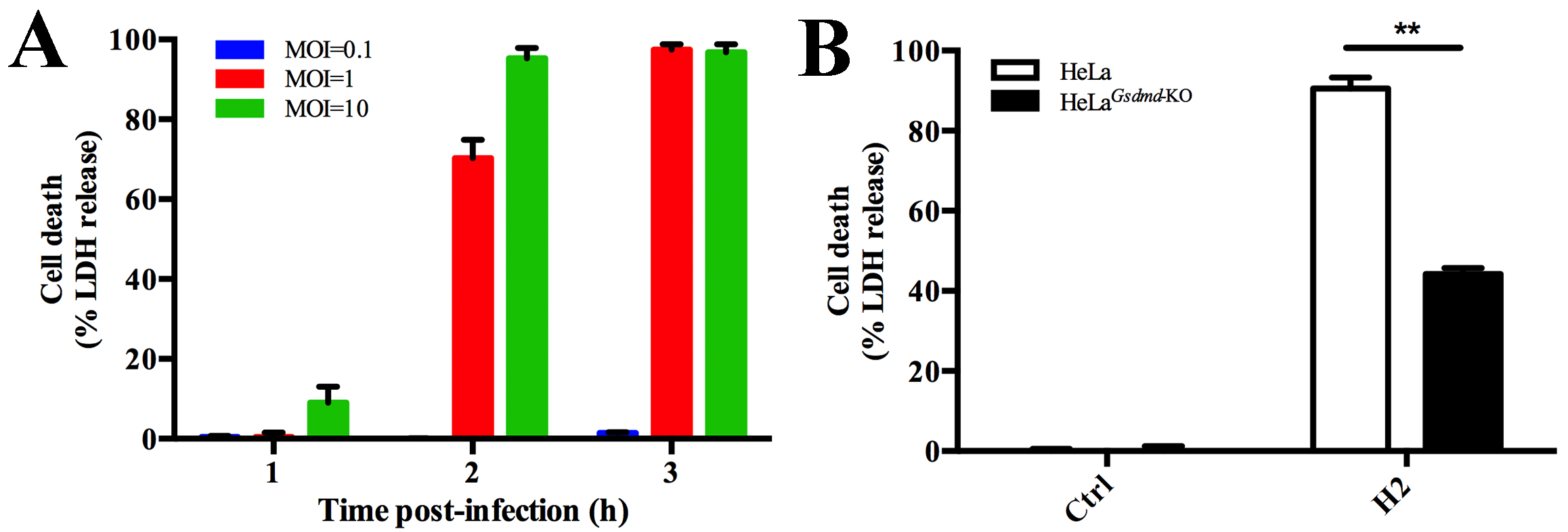

Supplement: Supplemental Material [file KVIR_A_1926649_SM3662.zip › supplementary(1926649)/Figure S4.tif]

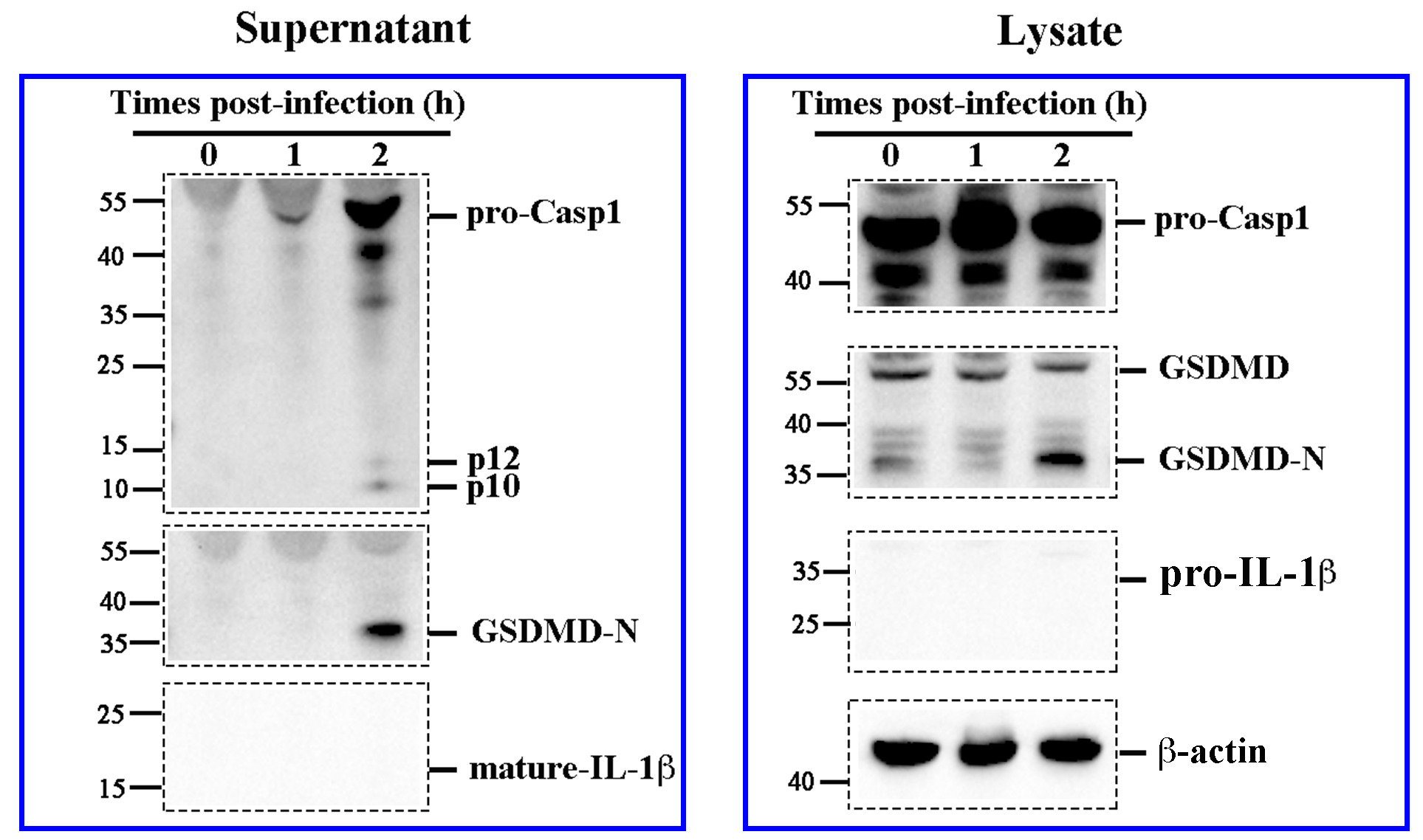

Supplement: Supplemental Material [file KVIR_A_1926649_SM3662.zip › supplementary(1926649)/Figure S5.tif]

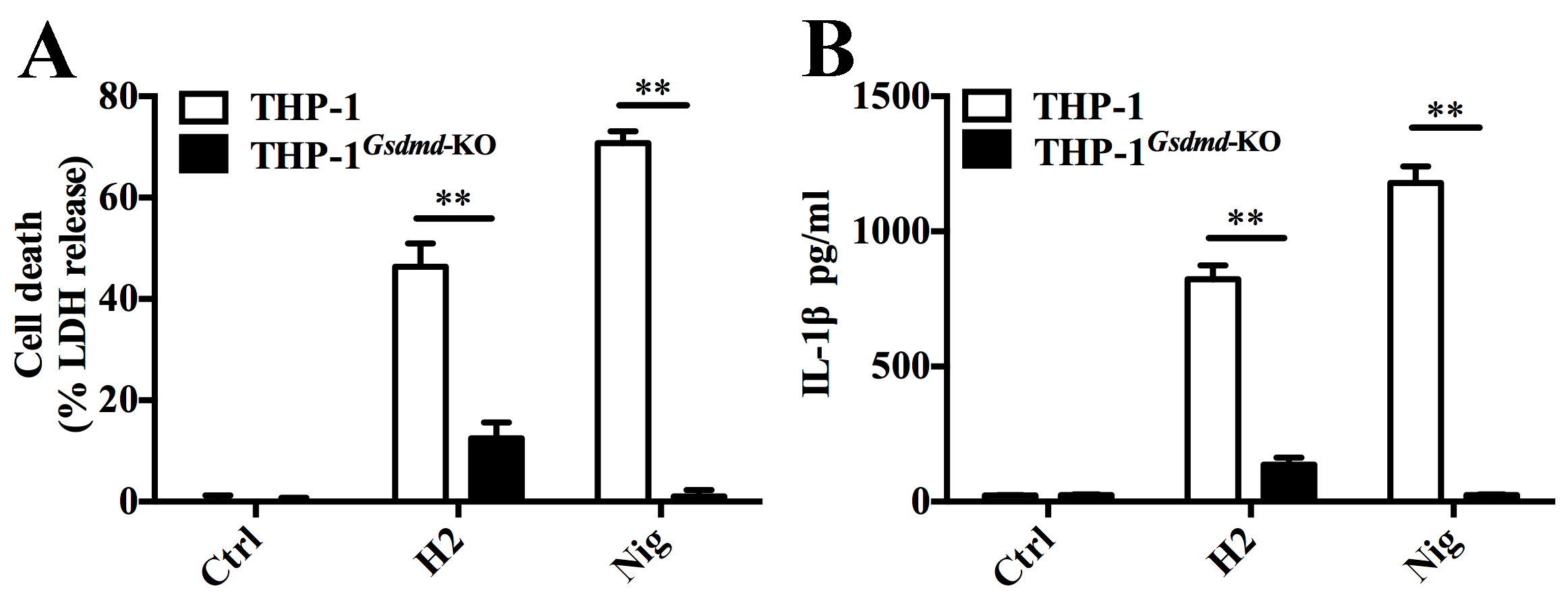

Supplement: Supplemental Material [file KVIR_A_1926649_SM3662.zip › supplementary(1926649)/Figure S6.tif]

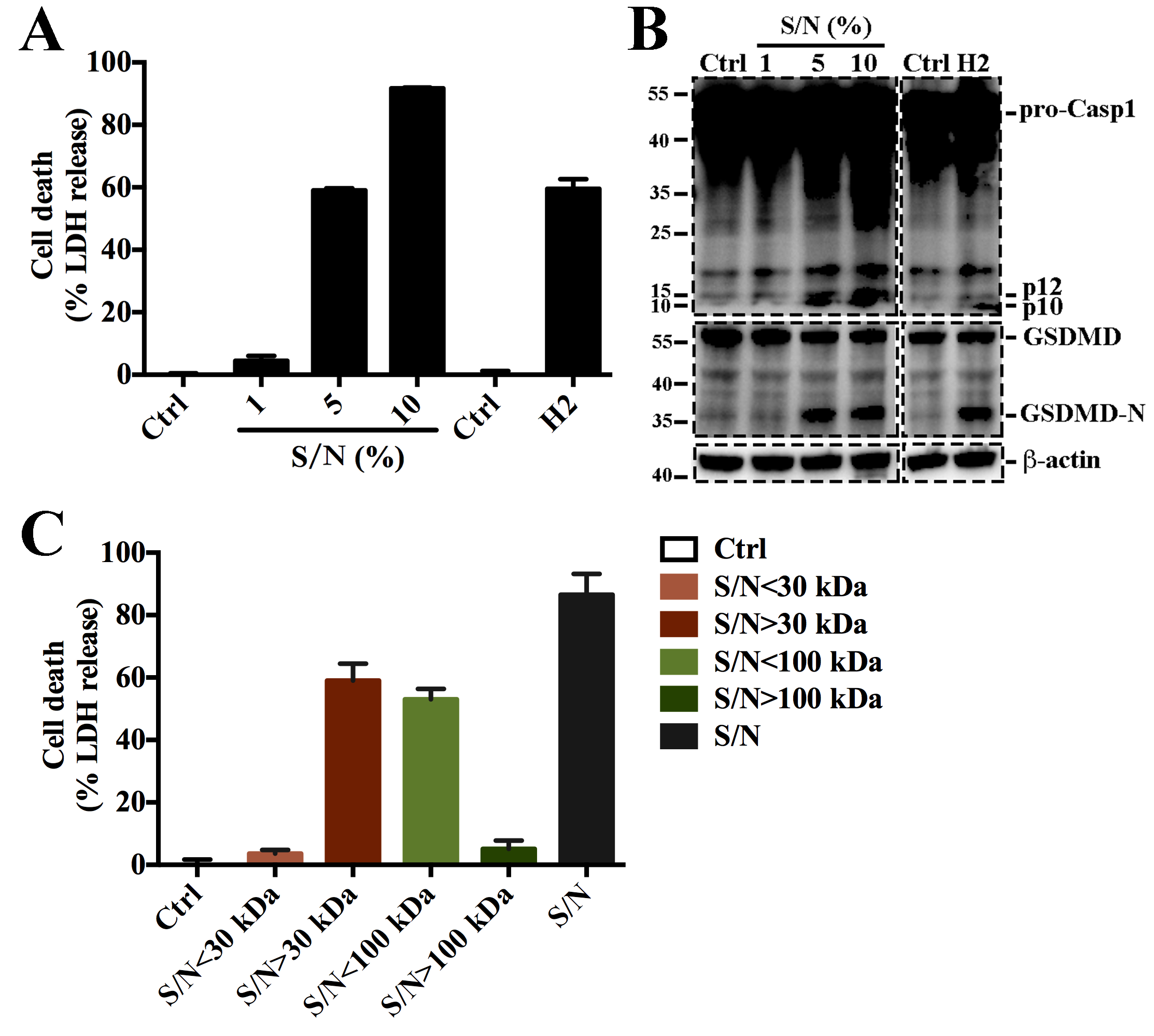

Supplement: Supplemental Material [file KVIR_A_1926649_SM3662.zip › supplementary(1926649)/Figure S7.tif]

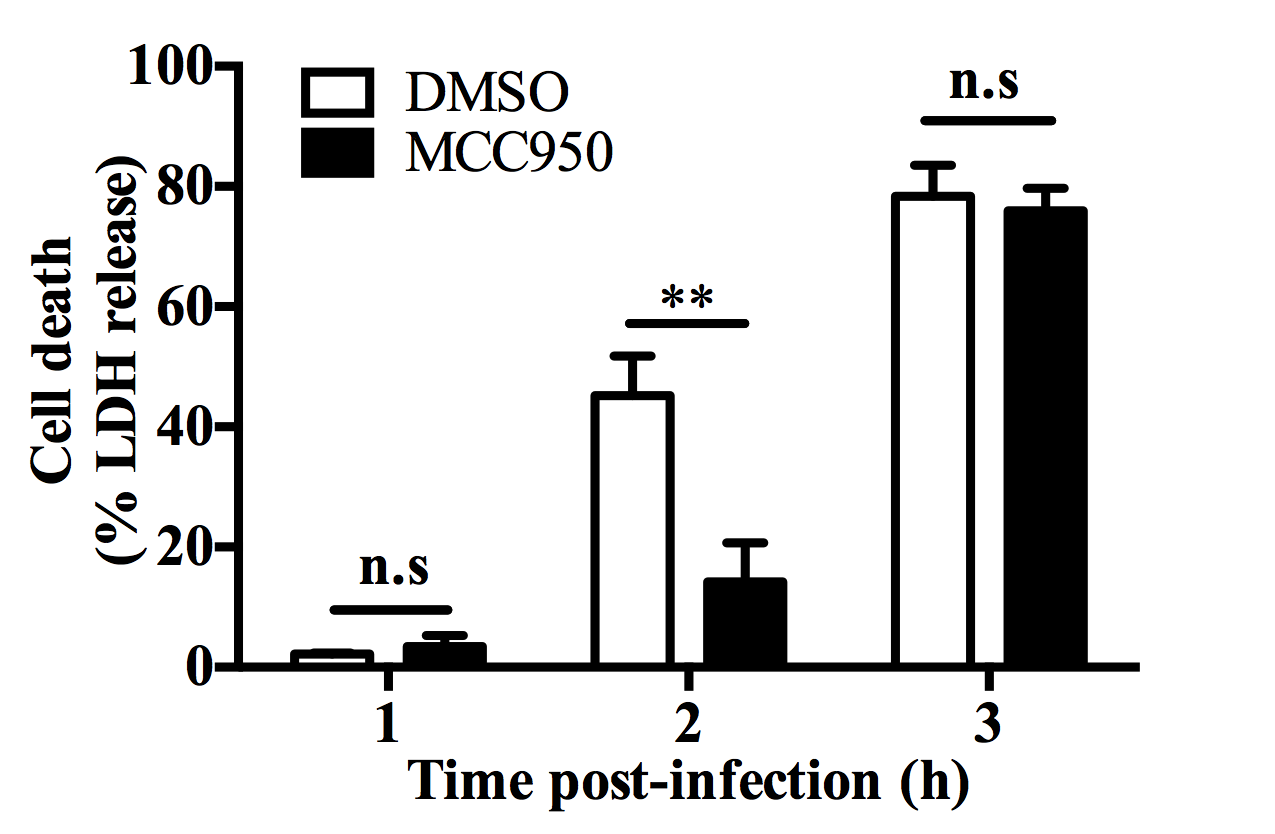

Supplement: Supplemental Material [file KVIR_A_1926649_SM3662.zip › supplementary(1926649)/Figure S8.tiff]

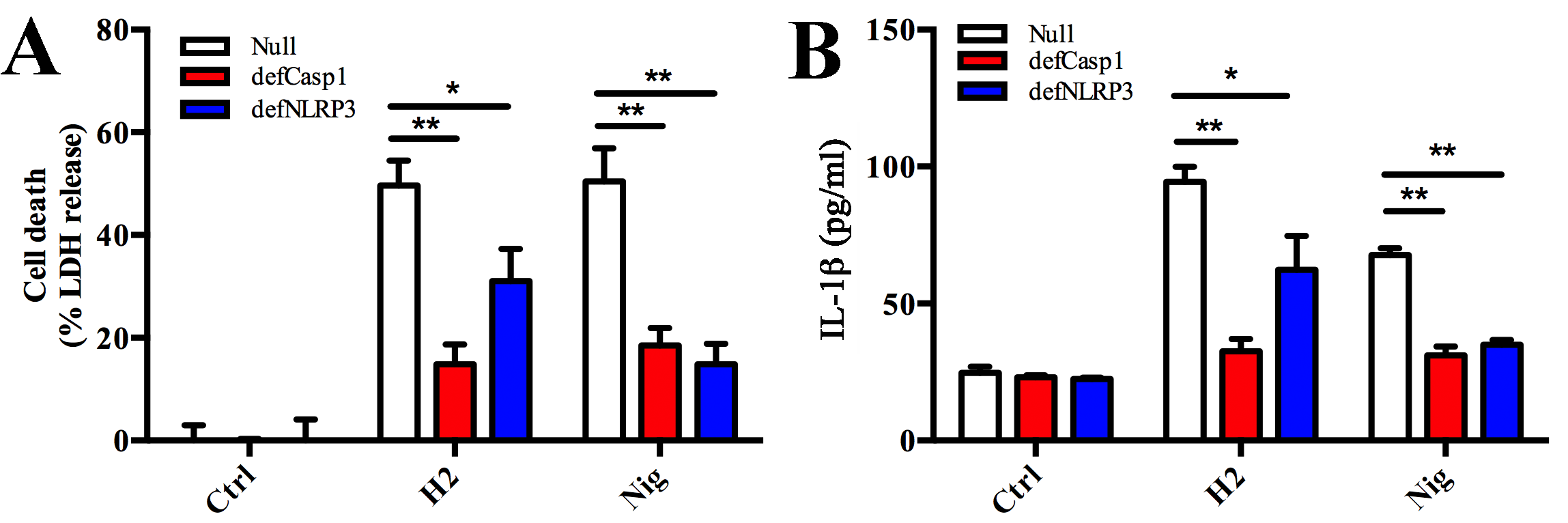

Supplement: Supplemental Material [file KVIR_A_1926649_SM3662.zip › supplementary(1926649)/Figure S9.tif]
